# Transactions of the Medical Association of Georgia

**Published:** 1866-07

**Authors:** L. H. Orme

**Affiliations:** Secretary


### EDITORIAL AND MISCELLANEOUS.

*Transactions of the Medical Association of Georgia.*

The annual meetings of the Medical Association of Georgia having been suspended by the war since April, 1861, assembled at the call of the President, Dr. J. T. Banks, of Griffin, at the City Hall, in Atlanta, on 21st June, 1866.

The President called the meeting to order, and the deliberations were commenced with prayer by Rev. Dr. Tucker, of Atlanta.

The Secretary being absent, on motion of Dr. F. O. Dannelly, Dr. R. C. Word was chosen Secretary, *pro tem.*

On motion of Dr. W. F. Westmoreland, Secretary was required to compile a list of all the members in attendance at the annual meetings of 1858 to 1861, inclusive—the books of the Association having been burned in the destruction of Atlanta—and report the same to this body at the afternoon session.

On motion of Dr. Logan, Dr. Jones, of Barbour county, Alabama, was invited to a seat in the Association.

On motion of Dr. Dannelly, the following preamble and resolution were adopted:

*Whereas,* The records of the Medical Association of Georgia were destroyed by the Federal forces during the occupation of the city of Atlanta, in 1864, and we have no entire roll of members,

*Resolved,* That each member of the Association now present report his name to the Secretary, so as to perfect the organization, and designate those who are rightfully entitled to participate in the deliberations of the body.

The following members reported and had their names recorded: Drs. J. T. Banks, J. F. Alexander, T. S. Powell, A. Means, W. C. Moore, E. J. Roach, J. M. Boring, W. F. Westmoreland, Eben Hillyer, F. O. Dannelly, J. C. Habersham, J. G. Westmoreland, J. P. Logan, R. C. Word, H. L. Wilson, Geo. G. Crawford, E. L. Connally, D. H. Connally and D. C. O'Keefe.

The Chair appointed as Committee on Credentials, Drs. Logan, Dannelly and J. G. Westmoreland.

On motion of Dr. W. F. Westmoreland, the regular order of business was suspended for the reception of new members; whereupon the following gentlemen were proposed, vouched for and elected members:

Drs. L. H. Orme, J. E. Godfrey, W. L. Armstrong, C. L. Redwine, H. L. Orme, D. W. Delbridge, B. M. Cromwell, J. C. Habersham and J. L. Moore.

On motion, Association adjourned till four p. m.

FOUR O'CLOCK, P. M.—The Association met pursuant to adjournment.

In accordance with resolution passed this morning, the Secretary *pro tem.* presented the required list of members recorded at the annual meetings alluded to.

Dr. Logan, Chairman of Committee on Credentials, made the following report, which was adopted:

The Committee on Credentials beg leave to report partially, that they have no information which would discredit any member of this Association, so far as their names have been recorded at this meeting. They would also solicit any information which may be in possession of members which would be appropriate to the object of their appointment.

Under a suspension of the regular order, the following applicants were elected members:

Drs. J. E. H. Ware, W. C. Askin, Charles Pinckney and W. A. Love.

On motion of Dr. J. G. Westmoreland, a committee of five were appointed to nominate suitable names to be balloted for to fill the various offices the ensuing year. Committee: Drs. J. G. Westmoreland, L. H. Orme, J. E. Godfrey, E. L. Connally and N. B. Drewry.

On report of nominating committee, the following elections were made:

Dr. A. Means, President; Dr. F. O. Dannelly, 1st Vice President; Dr. L. H. Orme, Recording Secretary; Dr. J. L. Moore, Corresponding Secretary.

On motion, meeting adjourned till nine o'clock to-morrow morning.

JUNE 22, NINE O'CLOCK, A. M.—Association called to order by the President.

Regular order suspended, and on motion of Dr. Dannelly, editors admitted to seats on the floor.

On motion, regular order suspended, and following new members admitted: Dr. S. H. Stout and Dr. R. Q. Stacey.

On motion, the election of Treasurer was had, and resulted in election of Dr. H. L. Wilson.

Drs. J. G. Westmoreland and Habersham committee to induct President elect to the chair.

The retiring President, Dr. Banks, addressed the Association, on leaving the chair, in a forcible and interesting manner.

Dr. Means, on taking the chair, entertained the Association for a few moments in his usually eloquent style.

On motion of Dr. Logan, the thanks of the Association were tendered the late President, Dr. Banks, for the efficient discharge of his duties as presiding officer.

Dr. Dannelly, Chairman of Committee to secure Orator for the session, reported that Dr. A. W. Griggs would deliver the annual address; for which six p. m. to-day, was set apart.

On motion of Dr. Dannelly,

*Resolved*, That Dr. J. G. Westmoreland be requested to assist the Recording Secretary in recording in suitable books the names of members, and transcribing into the same, the Constitution and By Laws of the Association. Also, in collecting the proceedings of the various meetings, and preserving in proper form all the available information pertaining to its history.

On motion of Dr. Dannelly,

*Resolved*, That an assessment of one dollar be imposed on each member of this Association, to defray current expenses for the ensuing year; and that the Corresponding Secretary be instructed to notify each member of this action of the body, and request that they forward the amount due under this resolution.

On motion, the Committee on Prize Essays reported, through the Chairman, Dr. Means, an Essay entitled to the prize of $50 00, on diptheria, with the motto "*Sis sub judice.*"

On motion of Dr. Alexander, the sealed letter accompanying the essay, and containing the same motto, was then opened, and found to contain the name of Dr. E. L. Gaillard.

On motion *Resolved*, That a copy of the report of Committee on Prize Essays, with the prize this day awarded, be forwarded to Dr. Gaillard.

Dr. Habersham, of Savannah, reported the "Georgia Medical Society" of that city, in a flourishing condition.

Dr. W. F. Westmoreland reported favorably of the Atlanta Medical Society.

Dr. Griggs reported the objects and progress of the Georgia and Alabama Medical Association, at West Point, Georgia.

Dr. Crawford reported the requirements, progress, etc., of Fulton County Medical Society.

On motion, meeting adjourned till three p. m.

THREE P. M.—Meeting called to order by the President.

On motion, the regular order was suspended, and Dr. J. E. Blackshear was elected member of the Association.

Dr. Dannelly entertained the Association for a short time on the subject of artificial limbs.

On motion of Dr. Logan,

*Resolved*, That the thanks of this Association be tendered Dr. J. T. Banks, the late President of this body, for the deep interest manifested in its success, resulting in its reorganization, and for the highly efficient manner in which he has discharged the duties of its presiding officer.

Dr. Habersham moved that the portion of the Constitution authorizing the admission of State licentiates to membership in the Association, be stricken out. After some discussion, on motion of Dr. Godfrey, the further consideration of the subject was postponed to the next meeting of the Association.

Dr. J. G. Westmoreland moved the appointment of a committee to memorialise the Legislature on registration of births and deaths. Committee: Drs. Habersham, J. G. Westmoreland and Word.

The balloting for the next place of meeting for the Association, resulted in the selection of Griffin.

On motion of Dr. Banks,

*Resolved*, That the permanent location of the Asociations at some suitable place, in the opinion of this meeting, is called for by its highest interests; and that in view of said interests, we do invite and call upon its members in every portion of the State, to meet with us at our next annual meeting, and settle definitely this question.

On motion of Dr. Dannelly,

*Resolved*, That a committee of three be appointed by the Chair to prepare an appropriate sketch of the life and character of those members of the Association who have died since our last meeting Committee: Drs. Dannelly, Banks and Logan.

On motion of Dr. O'Keefe, Drs. O'Keefe and W. F. Westmoreland were appointed a Committee on Essays.

On motion, Drs. Banks, J. G. Westmoreland, Dannelly, Drewry and O'Keefe were constituted a committee to examine prize essays.

On motion of Dr. O'Keefe, Dr. Banks was requested to furnish the Association with a copy of his address delivered on retiring from the chair.

On motion of Dr. J. G. Westmoreland,

*Resolved*, That the sum of one hundred dollars be hereby offered by the Association for the best prize essays—$50 for the best; $30 for the second, and $20 for the third.

The President read a letter from Dr. J. M. Johnson, President Atlanta Medical Society, which the Secretary was requested to answer.

On motion, regular order of business was suspended for the admission of new members, when Dr. J. M. Johnson was elected member.

On motion of Dr. Logan, the letter of Dr. Johnson was ordered spread upon the minutes.

On motion of Dr. Habersham, Drs. Habersham and Powell were made a committee to publish, as soon as funds can be obtained, the prize essay of Dr. Gaillard.

Drs. Darnall, Drewry, Moore and Banks were appointed on the Committee of Arrangements, and required to select an orator to deliver the next annual address.

The hour appointed for the annual address of the present session having arrived, the business of the Association was suspended for its delivery.

Dr. Griggs being conducted to the platform, entertained the Association for a short time with some pertinent and eloquent remarks touching subjects intimately connected with the interests of the medical profession.

On motion for adjournment, the President, Dr. Means, after making some touching allusions to our separation, and eloquent appeals in behalf of the honorable cause in which we are engaged, adjourned the Association to the next annual meeting in Griffin, on the second Wednesday in April, 1867.

L. H. ORME, Secretary.

